# CCR5 is a prognostic biomarker and an immune regulator for triple negative breast cancer

**DOI:** 10.18632/aging.203654

**Published:** 2021-10-30

**Authors:** Xin Wang, Yong Han, Jiamin Peng, Jie He

**Affiliations:** 1 Thoracic Surgery Department, National Cancer Center/National Clinical Research Center for Cancer/Cancer Hospital, Chinese Academy of Medical Sciences and Peking Union Medical College, Beijing, China; 2 Department of Thoracic Surgery, Zhejiang Provincial People’s Hospital, Affiliated People’s Hospital, Hangzhou Medical College, Hangzhou, Zhejiang, China; 3 Key Laboratory of Tumor Molecular Diagnosis and Individualized Medicine of Zhejiang Province, Zhejiang, China; 4 Department of Clinical Laboratory, Tongde Hospital of Zhejiang Province, Hangzhou, Zhejiang 310012, China

**Keywords:** CCR5, TNBC, multi-omics landscape, immune therapy, prognosis

## Abstract

This study aims to explore the clinical implications and potential mechanistic functions of CCR5 in triple negative breast cancer. Briefly, we demonstrated that CCR5 is overexpressed in TNBC and is associated with better prognosis of TNBC. CCR5 expression is positively correlated with tumor immune cell infiltration and tumor immune response related pathways. Multi-omics data analyses identified CCR5 associated genomic and proteomic changes. CCR5 overexpression was associated with better overall survival in TNBC patients with TP53 mutation. We also summarized the latest findings on ICB efficacy related genes and explored the association between CCR5 and those genes. These results indicated that CCR5 is a potential tumor suppressor gene and individualized therapeutic strategy could be established based on multi-omics background and expression pattern of ICB related genes. In conclusion, CCR5 is associated with better survival of TNBC patients with TP53 mutation, which may exert its roles through tumor immune environment.

## INTRODUCTION

Cancer caused approximately 10 million of deaths in the year 2020 worldwide [1]. With the development of new technologies and experimental methods and devices, numerous research advancement have been achieved in both basic and clinical fields in the last decade [2, 3]. Triple negative breast cancer (TNBC) is the most aggressive subtype of breast cancer with limited therapeutic options [4]. Recent studies revealed several underlying molecular mechanisms and potential drugs for TNBC [5, 6]. However, there is still a long way to go before clinical application. It is crucial to clarify the mechanisms underly TNBC’s aggressive phenotype and explore novel prognostic biomarkers and therapeutic targets.

It was reported that CCR5-Δ32/Δ32 genotype was associated with reduced life expectancy despite the protective effect of the mutation against HIV by analyzing genotyping and death registry information of 409,693 individuals of British ancestry [7]. This report indicated that CCR5 might have some positive roles in human fitness and thus caught the attention of the whole world immediately.

Actually, the function of CCR5 remain elusive despite intensive study in recent years. For instance, some researchers claimed that CCR5 could promote breast cad gastric cancer progression, while others demonstrated that the expression of CCR5 in both CD4+ and CD8+ T cells was critical in boosting anti-tumor immune response [8–11]. We and our collaborators showed that CCR5 overexpression was associated with better prognosis of breast cancer, lung cancer, liver cancer, rectal cancer and cervical cancer which might owing to its association with immune cell infiltration [12]. In this study, we will analysis the expression and prognostic value of CCR5 in TNBC and explore the underlying molecular mechanisms using multi-omics data. We will also discuss the association between CCR5 and ICB efficacy related genes and offer potential therapeutic options for TNBC.

## RESULTS

### CCR5 expression and its association with survival and TIL in basal-like or triple negative breast cancer

**Figure 1:**
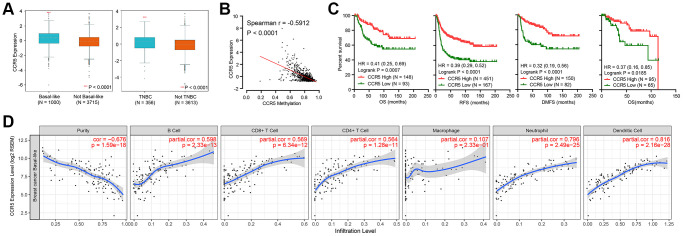
(**A**) CCR5 is overexpressed in basal-like or TNBC subtypes of breast cancer compared to not basal-like or non-TNBC subtypes. (**B**) The expression of CCR5 is negatively correlated CCR5 promoter methylation levels. (**C**) Patients with CCR5 high expression have better survivals compared to CCR5 low expression group. (**D**) CCR5 expression is positively correlated with tumor infiltration immune cells such as B cell, CD8+ T cell, CD4+ T cell, Neutrophil and Dendritic cell.

CCR5 is overexpressed in breast cancer in comparison to matched normal control (Supplementary Figure 1, *P* < 0.0001). Its expression level in basal-like breast cancer or TNBC are significantly higher than in non-basal-like or non-TNBC samples (Figure 1A, expression data is obtained from bc-GenExMiner database, *P* < 0.0001). Methylation level of CCR5 promoter region was significantly associated with CCR5 expression (Spearman r = 0.5912, *P* < 0.0001, Figure 1B), which meant that methylation might be one of the main regulators of CCR5 expression. As is shown in Figure 1C, Kaplan–Meier analyses indicated that CCR5 expression was positively correlated with overall survival (OS), recurrence free survival (RFS) and distant metastasis-free survival (DMFS) (merged TNBC gene expression data and corresponding clinical information were from KM plot, HR = 0.41, 0.39 and 0.32; *P* = 0.0007, <0.0001 and <0.0001, respectively), which was further confirmed by analyzing TCGA TNBC data (OS, HR = 0.37, *P* = 0.0185). Tumor immune cell infiltration analyses using TIMER in basal-like breast cancer samples showed that adjusted CCR5 expression was significantly correlated with tumor infiltration of B cell, CD8+ T cell, CD4+ T cell, Neutrophil and Dendritic cell (*P* < 0.0001), while there was no correlation between CCR5 expression and Macrophage infiltration (*P* = 0.233) (Figure 1D).

### GSEA analysis of CCR5 using 1570 breast cancer samples

**Figure 2:**
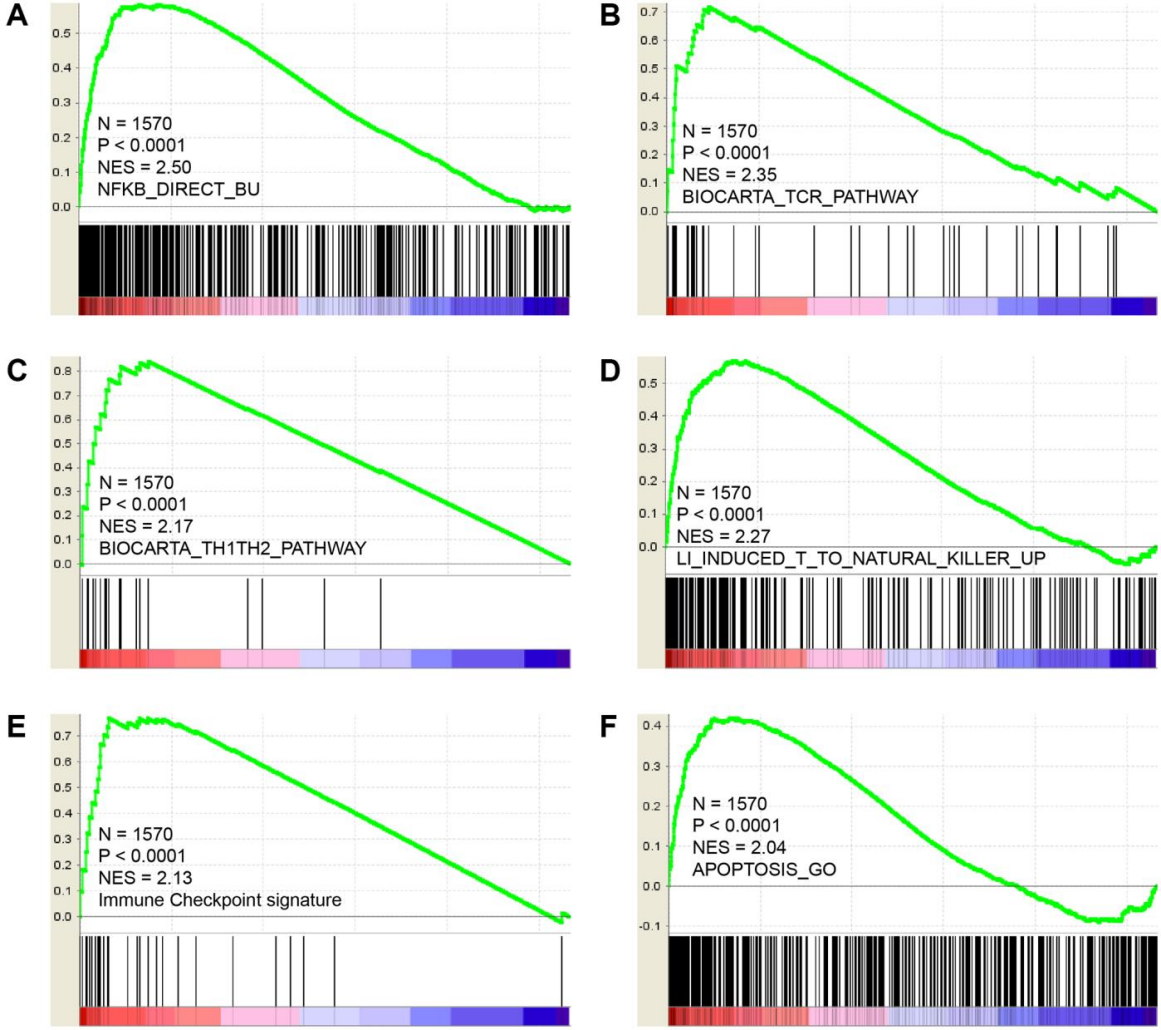
GSEA results show that CCR5 is positively correlated with NF-κB pathway (**A**), TCR pathway (**B**), TH1/TH2 pathway (**C**), Natural killer cell up (**D**), immune checkpoint signature (**E**) and Apoptosis (**F**).

Since CCR5 is overexpressed in TNBC/basal-like breast cancer and is associated with better survival and tumor immune cell infiltration, we wish to elucidate the potential roles of CCR5 in breast cancer using an expression profiling dataset containing 1570 breast cancer samples. GSEA analyses results (Figure 2) indicated that CCR5 expression was positively correlated with innate inflammation pathways such as NF-κB pathway (Figure 2A, NES = 2.5, *P* < 0.0001). It was also associated with TCR pathway, Th1Th2 pathway, T cell to Natural killer pathway and immune checkpoint related signatures (Figure 2B–2E, NES = 2.35, 2.17, 2.27, 2.13, respectively, all *P* < 0.0001). Moreover, CCR5 was associated with apoptosis (Figure 2F, NES = 2.04, *P* < 0.0001). These results indicated that CCR5 might repress breast cancer progression through NF-κB pathway, immune cell activation and pro-apoptosis.

### The genomic landscape of CCR5 high and low expressed TNBC patients

**Figure 3:**
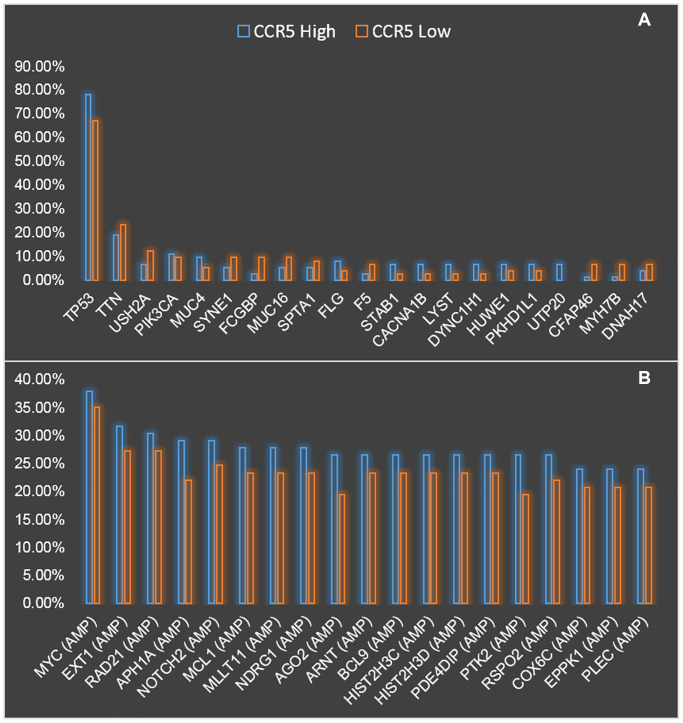
Comparison of mutation (**A**) and CNV (**B**) landscapes between CCR5 high expression and low expression groups.

Next, the genomic landscape of TNBC patients with CCR5 high and low expression were compared and presented in Figure 3. Figure 3A shows the comparison of mutation profiles between CCR5 high and low groups. We can see that TP53 has the highest mutation rate both in CCR5 high and CCR5 low TNBC patients (78.08% vs. 67.12%). Since the mutation rate of TP53 in breast cancer is around 30%, the high mutation rate of TP53 in TNBC indicates its potential role in TNBC progression. The following mutated genes are TTN, USH2A and PIK3CA etc. (19.18% vs. 23.29%, 6.85% vs. 12.33% and 10.96% vs. 9.59%, respectively). Figure 3B presents the comparison of CNA profiles between CCR5 high and low groups. The top three gene with copy number variation are MYC, EXT1 and RAD21 (Amplification, 37.97% vs. 35.06%, 31.65% vs. 27.27% and 30.38% vs. 27.27%, respectively). Detailed mutation and CNA profiling data comparison were presented in Supplementary Table 1.

### Prognostic value of CCR5 in TP53 wildtype/mutation TNBC patients

**Figure 4:**
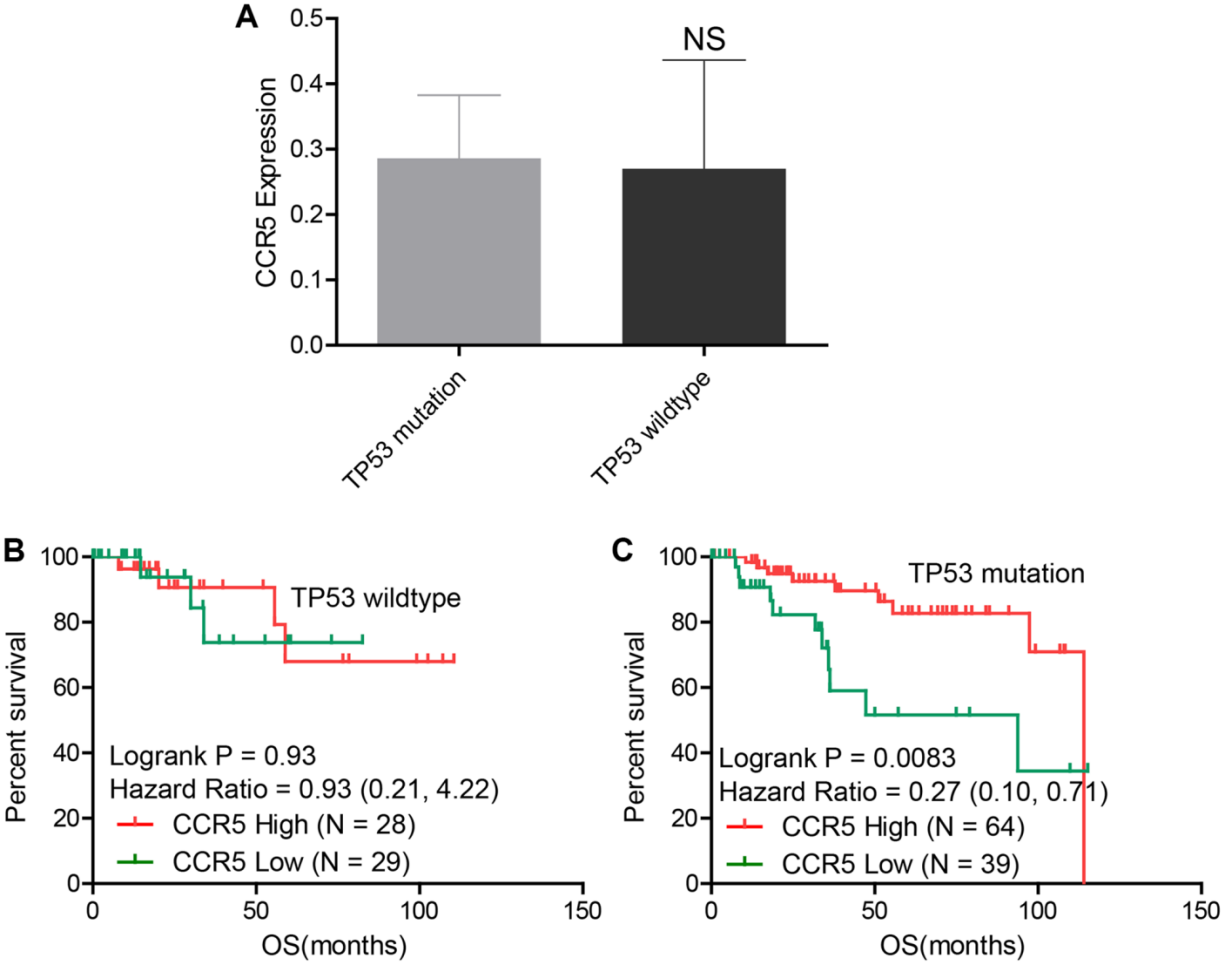
(**A**) There is no statistical difference of CCR5 expression between TP53 wildtype and TP53 mutation groups in TNBC samples. (**B**) CCR5 expression is not associated with OS in TNBC patients with wildtype TP53. (**C**) Patients with high CCR5 expression have better survival in TP53 mutation TNBC samples.

Results in the previous section showed that TP53 was highly mutated in TNBC, the association between CCR5 expression and TP53 mutation was explored in this part. Using expression and mutation data of TCGA, we demonstrated that there was no statistical difference of CCR5 expression in TNBC patient with or without TP53 mutation (Figure 4A). Kaplan-Meier analysis results showed that CCR5 expression is not associated with overall survival of TNBC patients in TP53 wildtype group (HR = 0.93, *P* = 0.93, Figure 4B), while high expression of CCR5 was associated with better overall survival in TNBC patients with TP53 mutation (HR = 0.27, Logrank *p* = 0.0083, Figure 4C). The above results indicate that CCR5 expression is not correlated with TP53 mutation status, however, the function of CCR5 in mitigating TNBC progression may rely on TP53 mutation. We tried to explain this interesting phenomenon by analysis the expression pattern of CCR5 and immune checkpoint markers in TNBC patients with or without P53 mutation (Supplementary Figures 2–3). The correlation between CCR5 expression and infiltrated immune cells were also visualized in Supplementary Figures 4–5.

### Proteomic analysis of CCR5 high and low expressed TNBC patients

**Figure 5:**
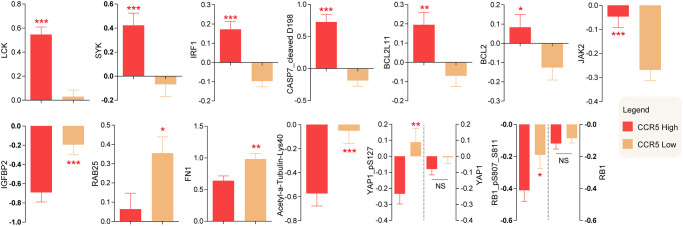
Differentially expressed proteins between CCR5 high expression and low expression groups.

Reverse phase protein array (RPPA) data of TNBC, which containing 226 antibodies, was downloaded from TCGA. Differentially changed proteins were computed and top changed proteins were presented in Figure 5 (For detailed information, please see Supplementary Table 1). LCK, SYK, IRF1, CASP7_cleaved D198, BCL2L11, BCL2 and JAK2 were higher in TNBC patients with high CCR5 expression, while IGFBP2, RAB25, FN1, Acetyl-α-tubulin-Lys40, YAP1_pS127, RB1_pS807_S811 were lower in CCR5 high TNBC patients. There are no statistical differences of YAP1 and RB1 between CCR5 high and CCR5 low TNBC patients. These proteomic changes might partly account for CCR5 associated good prognosis.

### Clustering analysis of CCR5 and immune checkpoint related signatures in TNBC samples

**Figure 6:**
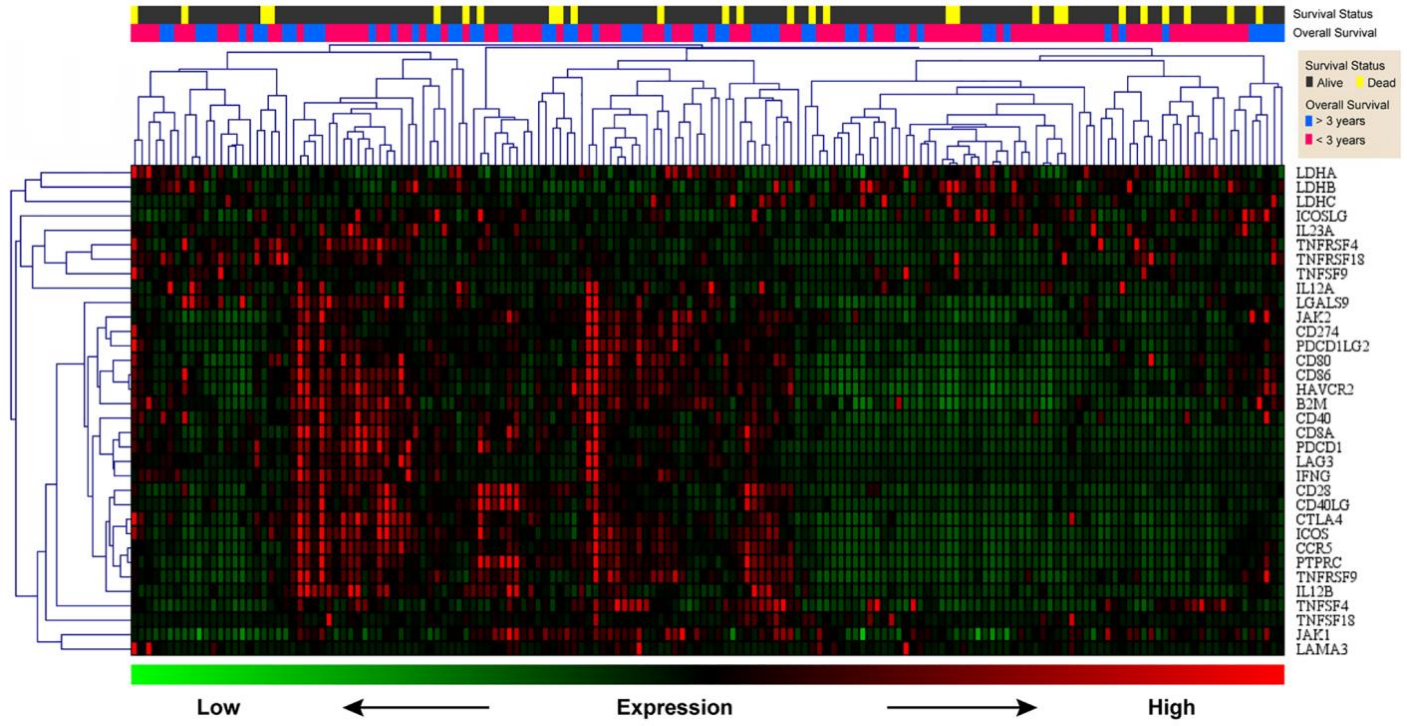
**Heat map of CCR5 and immune checkpoint related genes.** Green represents low expression level and red represents high expression level. The two upper rows represent survival and survival status, respectively.

**Figure 7:**
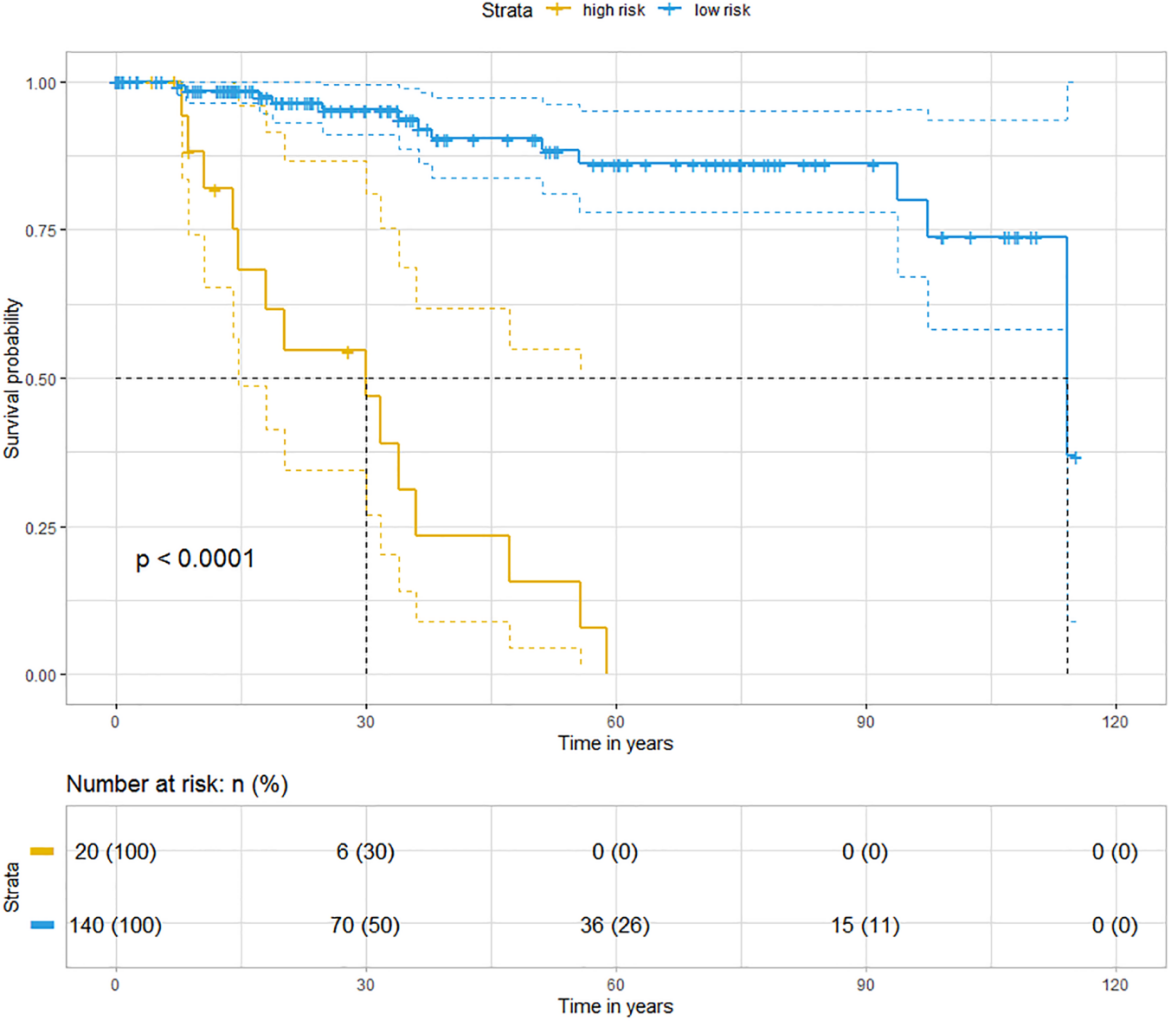
Kaplan-Meier analyses indicate that patients in low risk group have better OS than those in high risk group (blue represents low risk group and yellow represents high risk group, Logrank *p* < 0.0001).

Previously, we demonstrated that CCR5 expression was correlated with tumor immune cell infiltration. Since TILs were often associated with the efficacy of immunotherapy, the prognostic of CCR5 in immunotherapy would be very interesting. Here, we performed ROC curve analyses using gene expression data of melanoma treated with immune checkpoint inhibitor (GSE91061, pre-treatment and on-treatment) to show the predictive power of CCR5 in predicting disease control rate (DCR). The AUC of pre-treatment CCR5 value (pre), on-treatment CCR5 value (on) and ‘on-treatment minus pre-treatment CCR5 value (on-pre)’ were 0.5509, 0.7338 and 0.7546, respectively (Supplementary Figure 6). The prognostic power of on-pre was the highest (sensitivity = 75%, specificity = 77.78%). These results showed that CCR5 elevation after immune checkpoint blockade (ICB) was a significant prognostic marker, which might also have therapeutic implications. Since CCR5 is associated with ICB efficacy, heat map and cluster analysis were performed using TNBC data from TCGA through MeV to show the expression pattern of CCR5 and immune checkpoint related genes. Figure 6 shows that these genes are clustered into three main groups: Lactate dehydrogenase (LDH); ICOSLG, IL23A, TNFRSF4, TNFRSF18, TNFSF9 and IL12A; CCR5 and other ICB efficacy related genes including PDCD1 (PD1), CD274 (PDL1), JAK2 and LAG3. Specifically, CCR5 expression pattern is more similar to PTPRC, TNFRSF9, ICOS and CTLA4. The above results indicate that CCR5 is a prognostic marker for ICB treatment and could serve as a potential therapeutic target. Cox regression for survival analysis using CCR5 and immune checkpoint related genes was performed. Risk groups were divided using best cutoff point of risk score. Figure 7 indicated that patients in low risk group had better OS than high risk group.

### Association analyses of CCR5 and ICB efficacy related genes in TNBC samples

**Figure 8:**
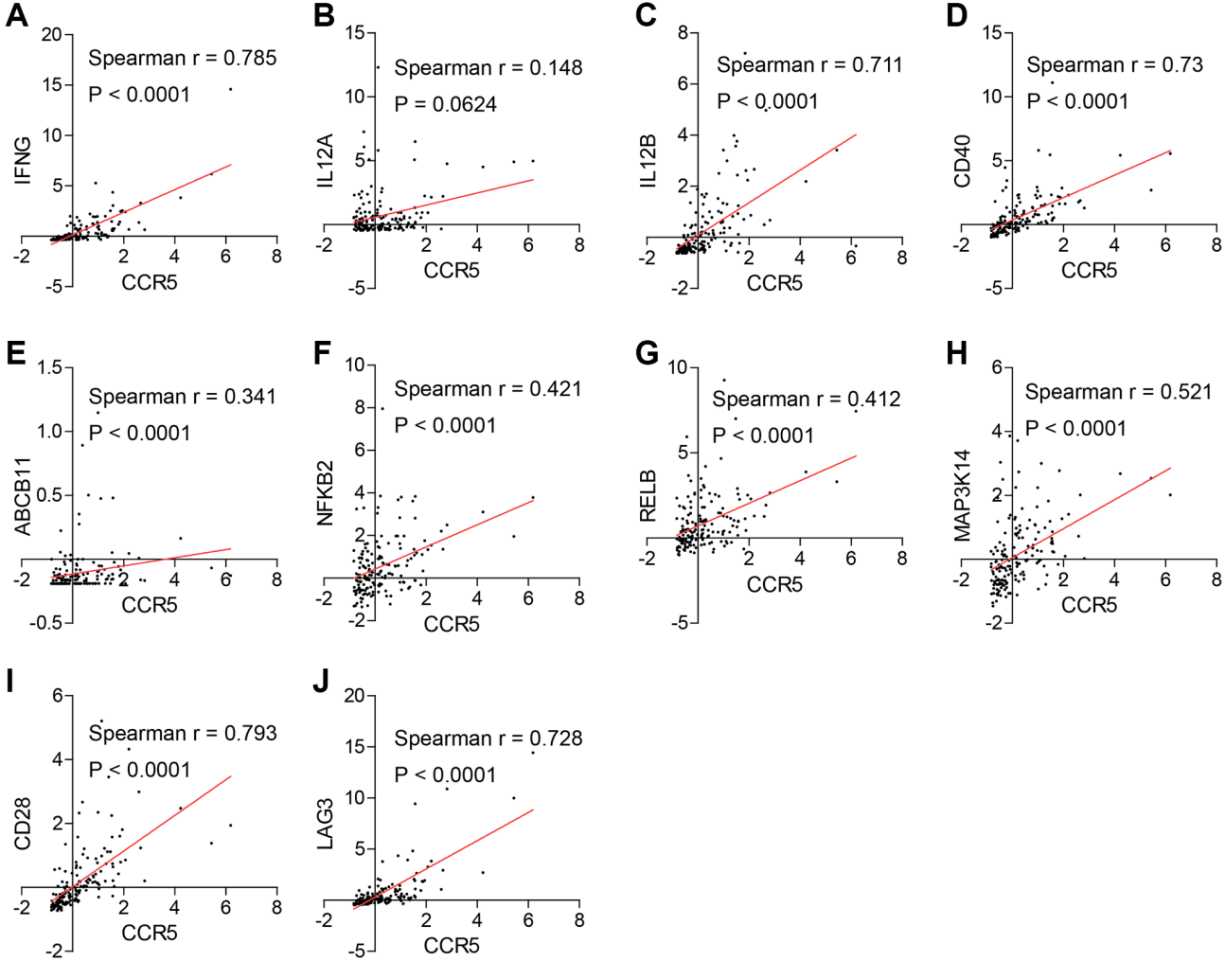
(**A**, **C**–**J**) CCR5 is positively correlated with good prognostic markers of anti-immune checkpoint therapies such as IFNG, IL12B, CD40, ABCB11, NFκB2, RELB, MAP3K14, CD28 and LAG3. (**B**) The correlation between the expression of CCR5 and IL12A does not have statistical significance (*p* = 0.0624).

Next, we discussed the latest findings on molecular basis of ICB resistance and analyzed the association between CCR5 and those genes. We demonstrate that in TNBC patients CCR5 is positively correlated with IFN-γ, IL12B and key non-canonical NF-kB pathway genes, namely CD40, ABCB11, NFKB2, RELB and MAP3K14 (Figure 8A and Figure 8C–8H). Besides, CCR5 is also positively correlated with T cell co-stimulator CD28 and immune checkpoint receptor LAG3 (Figure 8I–8J). While the correlation between CCR5 and IL12A does not have statistical significance (Figure 8B). FGL1 is overexpressed in breast cancer in comparison to matched normal control (Supplementary Figure 7A, *P* = 0.0039), but there is no statistical difference between basal-like and non-basal-like group of breast cancer (Supplementary Figure 7B). The correlation between CCR5 and FGL1 does not have statistical significance (Supplementary Figure 7C). SIGLEC15 and YTHDF1 are overexpressed in breast cancer and especially non-basal-like subtype (Supplementary Figures 8A–8B and 9A–9B). There is no correlation between the expression of CCR5 and SIGLEC15, YTHDF1 (Supplementary Figures 8C and 9C, *P* = 0.347 and 0.422, respectively). The above results indicate that in TNBC patients with CCR5 overexpression, T Cell-DCs crosstalk involving IFN-γ and IL12 is activated, which means these patients would show better therapeutic efficacy to anti-PD1 immunotherapy. Since the expression of SIGLEC and YTHDF1 are higher in not basal-like breast cancer subtype, these genes might be more promising therapeutic targets for non-basal-like subtype or breast cancer patients with low CCR5 expression.

## DISCUSSION

Great success has been achieved in cancer management in recent years. For instance, use implanted 3D-Printed vertebral bodies with robotic stereotactic radiotherapy for spinal tumor treatment [13]; encapsulate irinotecan (CPT-11) into micelle-based nanoparticles for a better efficacy in cancer therapy [14]; immunotherapy using immune checkpoint inhibitors has been applied in several solid tumors including TNBC [15, 16]; development of allele-specific K-RasG12C inhibitors for the treatment of oncogenic KRAS mutant in different cancer types [17]. However, the current situation is far from satisfied. It is still crucial to clarify the molecular mechanisms underly cancer progression and explore novel prognostic biomarkers and therapeutic targets.

Despite intensive studies on CCR5 in recent years, the roles of CCR5 in cancer remain elusive. Previously, we and our collaborators demonstrated that CCR5 is associated with better overall survival of several cancer types including breast cancer [12]. However, the expression pattern and prognostic value of CCR5 in different breast cancer subtypes and underlying mechanistic insights still needs to be clarified.

In this study, we showed that CCR5 is overexpressed in TNBC compared to non-TNBC or normal control and is associated with better prognosis of TNBC. CCR5 expression is positively correlated with tumor immune cell infiltration and tumor immune response related pathways. Multi-omics data of TNBC were compared based on CCR5 expression levels and CCR5 associated genomic and proteomic changes were identified. CCR5 overexpression was associated with better OS in TNBC patients with TP53 mutation. We also summarized the latest findings on ICB efficacy related genes and explored the association between CCR5 and those genes. These results indicate that CCR5 is a potential tumor repressor gene and individualized therapeutic strategy could be established based on multi-omics background and expression pattern of ICB related genes. Finally, several drugs that could potentially upregulated CCR5 expression were suggested.

Previously, Pestell et al. reported that CCR5 antagonists maraviroc could reduce invasion and metastasis of basal breast cancer cells *in vitro* and *in vivo*. And they suggested that CCR5 antagonists could be used as a therapeutic option for mitigating the risk of metastasis in patients with the basal breast cancer subtype [8]. Our results indicated that CCR5 is associated with longer OS of TNBC patients with TP53 mutation and is positively correlated with tumor immune response. These results seem to be inconsistent with Pestell’s results, which could be explained by drawbacks of their experiment design that merely using cancer cell lines and immunodeficient (NOD/SCID) mice model. Moreover, our study is supported by another group from The Scripps Research Institute, which showed that CCR5 expression in both CD4+ and CD8+ T cells was necessary to activate cancer immune response that might have implications for cancer treatment in patients with CCR5 deficiency.

We also summarized the latest findings on the molecular basis of ICB efficacy and discussed the association between CCR5 and those signatures. It is reported that effective anti-PD1 therapy requires the crosstalk between T cells and intratumoral dendritic cells (DCs) [18]. Specifically, anti-PD1 mAb could activate T cells and induce IFN-γ production, which further induced IL-12 production by DCs. Effective anti-PD1 therapy requires IL-12 produced by DCs to license effector T cell responses in cancer patients. Agonizing non-canonical NF-κB pathway can induce IL-12 production through DCs activation and enhance anti-PD1 therapy. Moreover, IL-12 and T cell co-stimulator CD28 are required to achieve maximal IFN-γ response. Lieping Chen et al. reported in *Cell* [19] that FGL1 was a major immune inhibitory ligand of LAG-3 and blockade of the FGL1-LAG-3 interaction could enhance anti-tumor immunity, suggesting that FGL1-LAG-3 pathway was an important immune evasion mechanism and a potential target for immunotherapy. Three months later, Lieping Chen group identified SIGLEC15 as a critical immune suppressor and its expression was mutually exclusive to PDL1, which implicated its potential therapeutic value in cancer patients especially for those who failed to response to anti-PDL1 therapy [20]. Chuan He et al. reported a novel immune evasion mechanism in *Nature* that the N6-methyadenosine (m6A) marked transcripts encoding lysosomal proteases were recognized and bounded by YTHDF1 in DCs, which promoted translation of lysosomal proteases for excessive neoantigen degradation, thereby mitigating neoantigen-specific tumor immunity. Furthermore, the efficacy of anti-PDL1 therapy was enhanced in Ythdf1−/− mice, suggesting YTHDF1 as a promising target for immunotherapy [21]. Here, we show that CCR5 is positively correlated IFN-γ, IL12B and key non-canonical NF-kB pathway genes such as CD40, ABCB11, NFKB2, RELB and MAP3K14. It is also associated with CD28 and LAG3, while there are no correlation between CCR5 and IL12A, FGL1, SIGLEC15 and YTHDF1. These results suggest that CCR5 may increase the efficacy of anti-PD1 therapy through activating T cell-DCs crosstalk.

Furthermore, we find several drugs that can upregulated CCR5 expression by exploring CTD database. For instance, Cisplatin, cyclophosphamide, Oxaliplatin, Topotecan and Clofibrate can promote CCR5 expression, which implicates their potential application in managing TNBC patients with low CCR5 expression (Supplementary Figure 10).

In summary, CCR5 is overexpressed in TNBC and is associated with better prognosis of TNBC with TP53 mutation. Potential mechanisms may include activation of certain tumor suppressors while repressing some oncogenic pathways such as YAP1. Activation of effector T cell may also account for CCR5 related tumor immune response. All these data suggest that CCR5 is a prognostic marker and potential therapeutic target for TNBC with TP53 mutation. Further wet lab experiments and clinical trials are warranted.

## MATERIALS AND METHODS

### Ethics statement

All the data used in this study were downloaded from publicly available sources. The Research Ethics Committee of Zhejiang Provincial people’s Hospital and National Cancer Center/National Clinical Research Center for Cancer/Cancer Hospital waived the requirement for ethical approval.

### Data source

Gene expression data for non-basal-like/basal-like or non-TNBC/TNBC comparison were obtained from bc-GenExMiner database. Gene expression data of CCR5 and other immune related genes in TNBC were downloaded from The Cancer Genome Atlas (TCGA: http://cancergenome.nih.gov/). Mutation, Methylation, Protein expression and copy number alteration data of TNBC were also obtained from TCGA. Data for survival analyses were downloaded from KMplot [22] and TCGA. All other expression data were obtained from Gene Expression Omnibus (GEO) [23]. Specifically, GSE70947 [24] was used for comparing expression levels between breast cancer and paired normal control; GSE47561 [25] (*N* = 1570) was used for Gene Set Enrichment Analysis (GSEA) [26]; GSE96061 [27] was used for ROC (Receiver operating characteristic) curve analysis. Chemical-gene interaction data was downloaded from The Comparative Toxicogenomics Database (CTD base) [28]. The abundance data of infiltrated immune cells in TCGA TNBC data was obtained from xCell [29].

### Bioinformatics and statistical analyses

Heat map and clustering analysis were performed using MeV software (http://mev.tm4.org). GSEA was performed to show the functional enrichment of CCR5 in breast cancer. Immune infiltration analysis was using TIMER [30]. R 4.0.0 (R Foundation for Statistical Computing (http://www.r-project.org/)) or GraphPad Prism 5.01 (GraphPad Software, Inc. (http://www.graphpad.com)) were utilized to perform all other statistical analyses. Cox regression and related survival analysis were performed using ‘survival’ and ‘survminer’ packages [31, 32]. Correlation analysis and visualization were performed using ‘corrplot’ package [33]. Standard statistical tests including paired *t*-test, fisher exact test and independent samples *t*-test were employed in the data analyses. Adjust *P* value was corrected for multiple comparisons using the Benjamini and Hochberg's false discovery rate [34]. Significance was defined as a *P* value < 0.05.

### Availability of data

Data sharing is not applicable to this article as no new data were created or analyzed in this study.

## Supplementary Materials

Supplementary Figures

Supplementary Table 1
