# Supplementary material for: CCR5 is a prognostic biomarker and an immune regulator for triple negative breast cancer
Source: Aging (Albany NY). 2021 Oct 30;13(20):23810–30. doi: 10.18632/aging.203654 (PMC8580338; doi:10.18632/aging.203654)
Supplement: Supplementary Figures [file aging-13-203654-s001.pdf]

SUPPLEMENTARY FIGURES

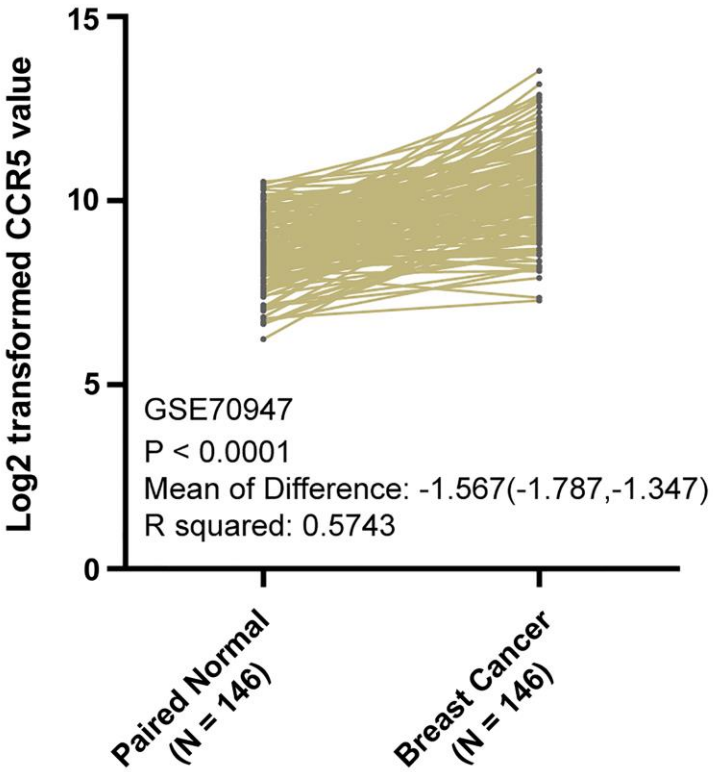

Supplementary Figure 1. CCR5 is overexpressed in breast cancer compared to matched normal control.

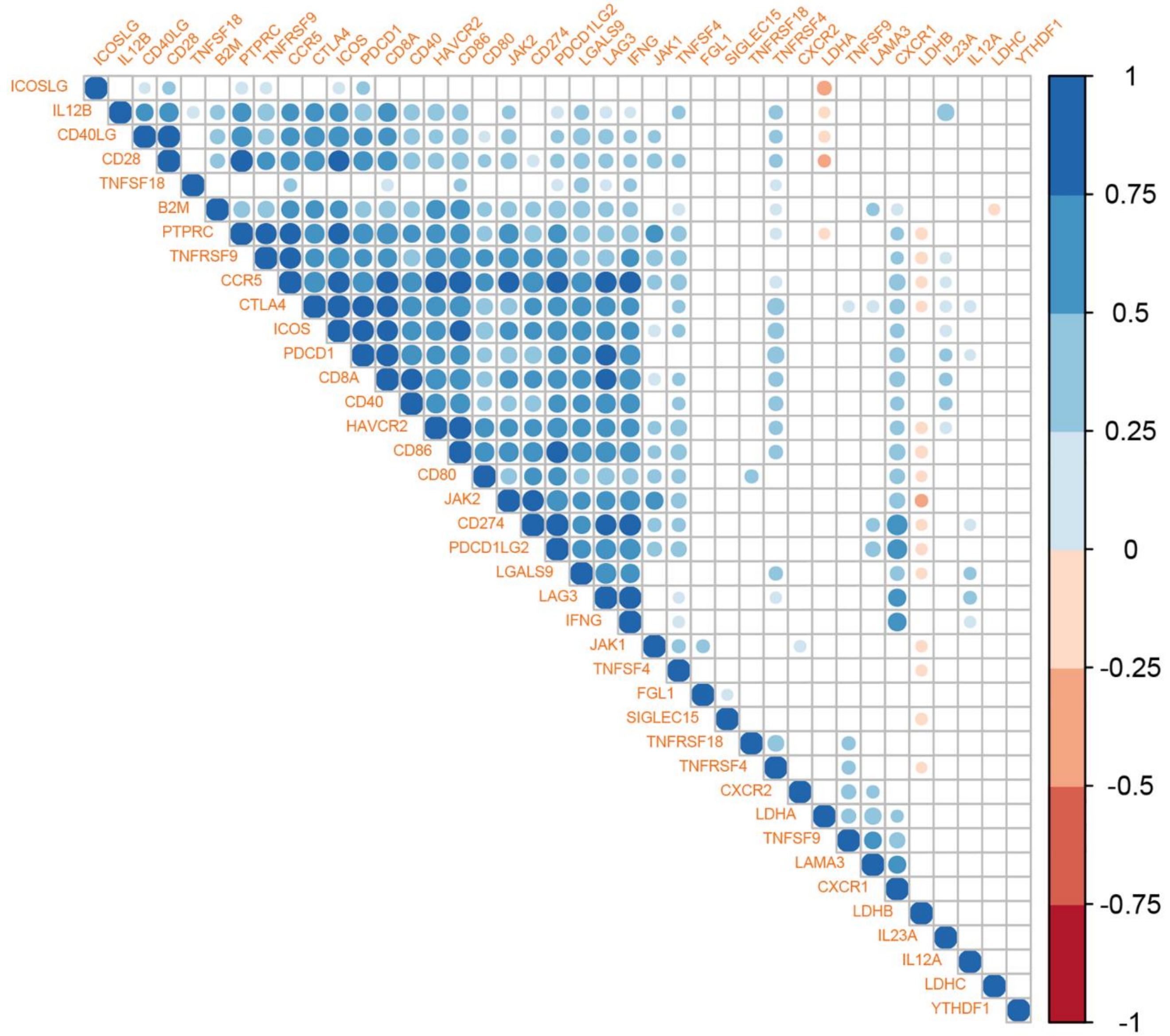

Supplementary Figure 2. The correlation between CCR5 expression and immune checkpoint markers in TNBC patients with P53 mutation.

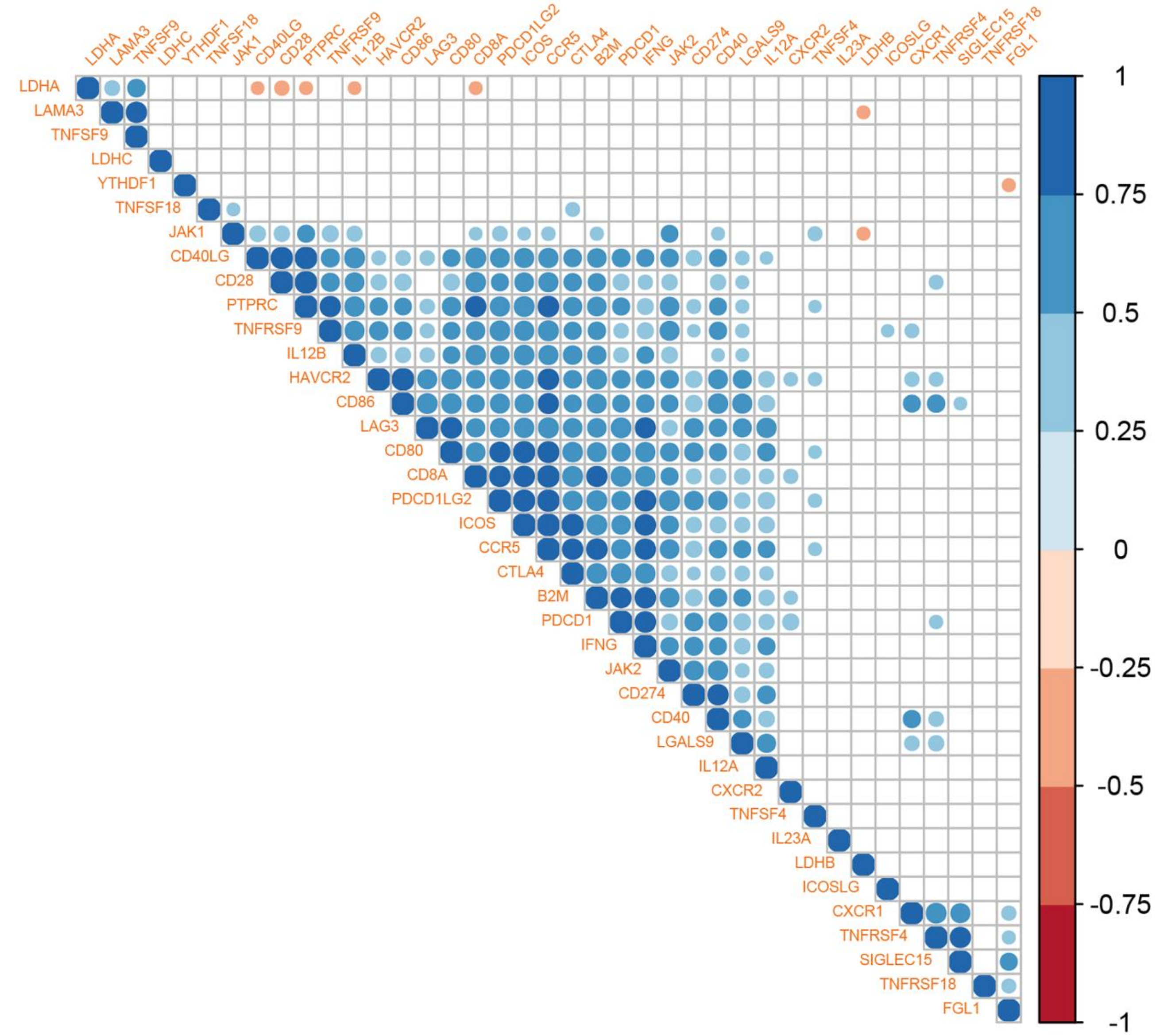

Supplementary Figure 3. The correlation between CCR5 expression and immune checkpoint markers in TNBC patients with wildtype P53.

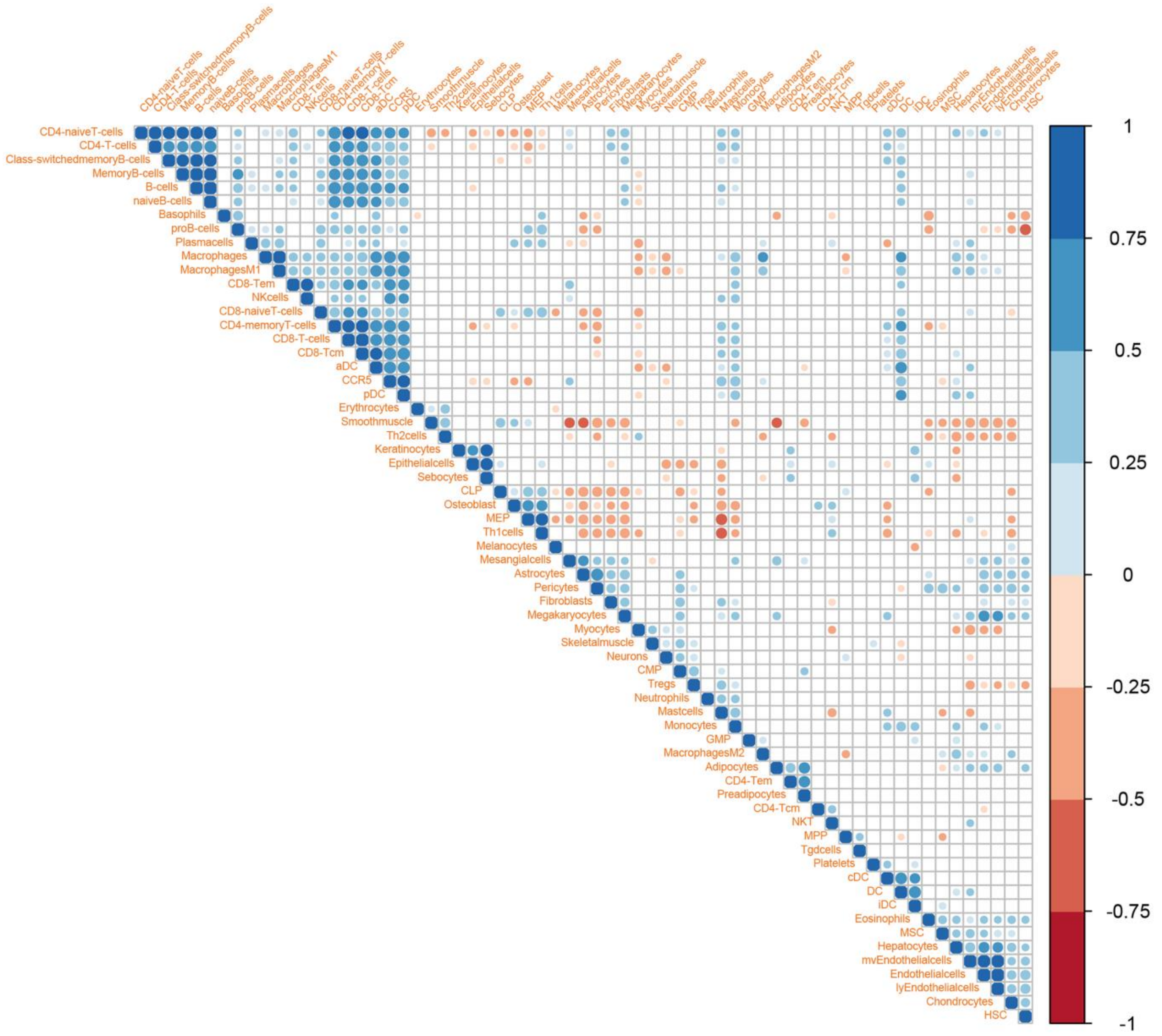

Supplementary Figure 4. The correlation between CCR5 expression and the abundance of tumor infiltrated immune cells in TNBC patients with P53 mutation.

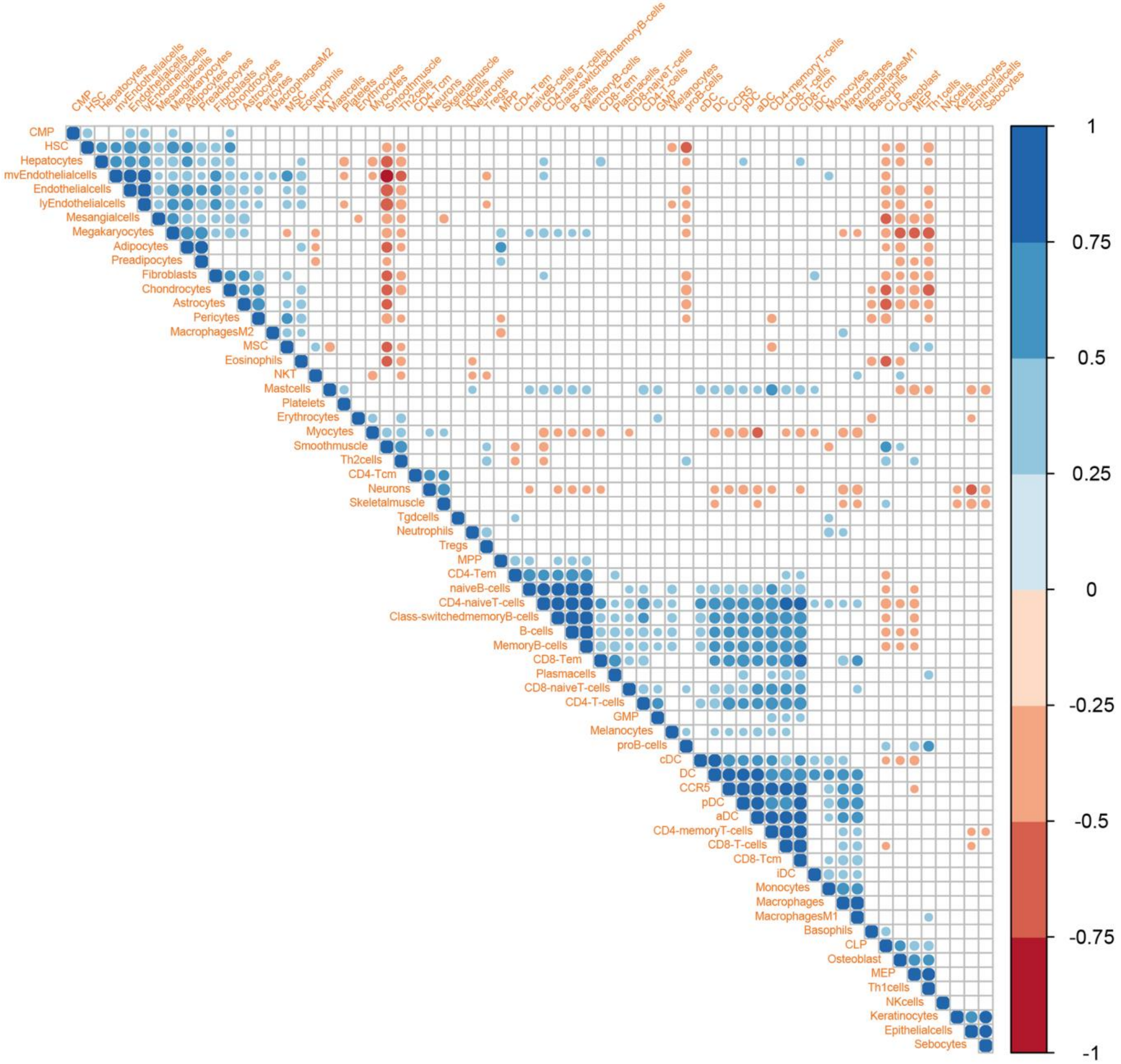

Supplementary Figure 5. The correlation between CCR5 expression and the abundance of tumor infiltrated immune cells in TNBC patients with wildtype P53.

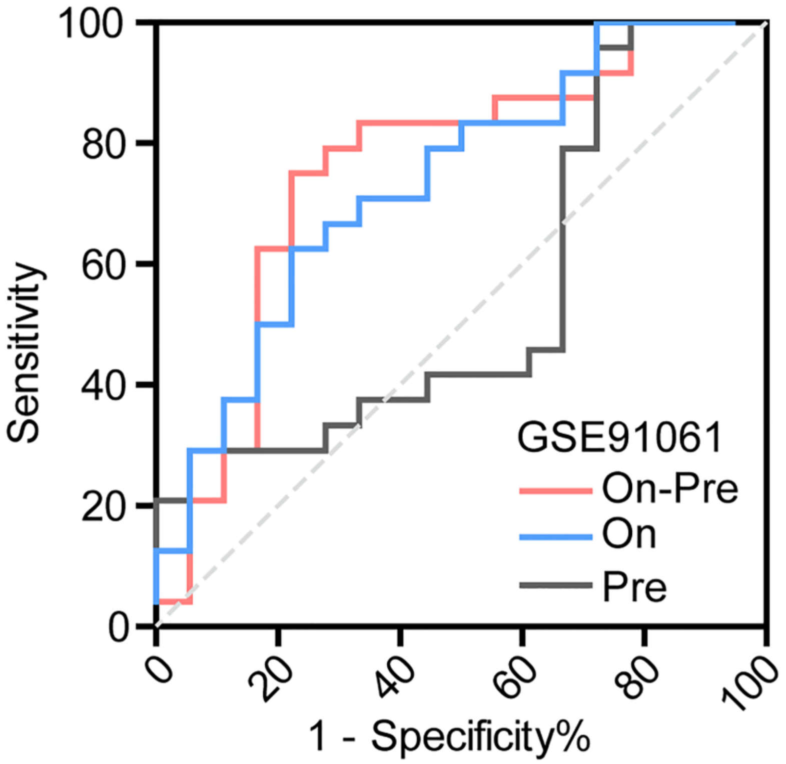

Supplementary Figure 6. ROC curves of CCR5 in pre-treatment, on-treatment and on-treatment minus pre-treatment. AUC are 0.5509, 0.7338 and 0.7546, respectively.

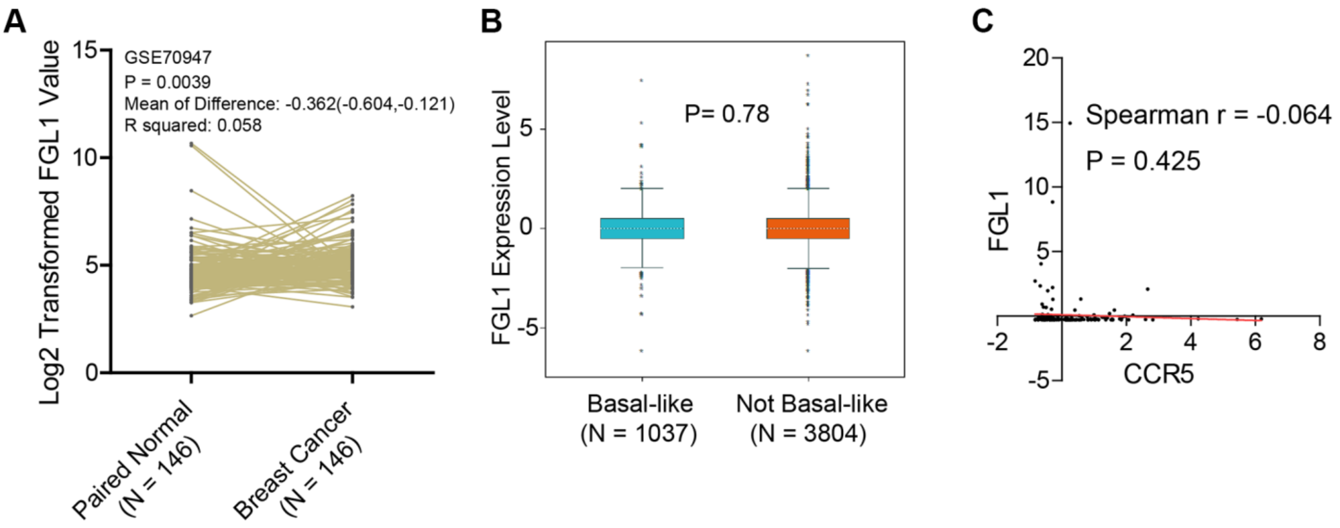

Supplementary Figure 7. (**A**) FGL1 is overexpressed in breast cancer compared to matched normal control. (**B**) There is no statistical difference of FGL1 expression between basal-like subtype and not basal-like subtypes. (**C**) There is no correlation between the expression of CCR5 and FGL1.

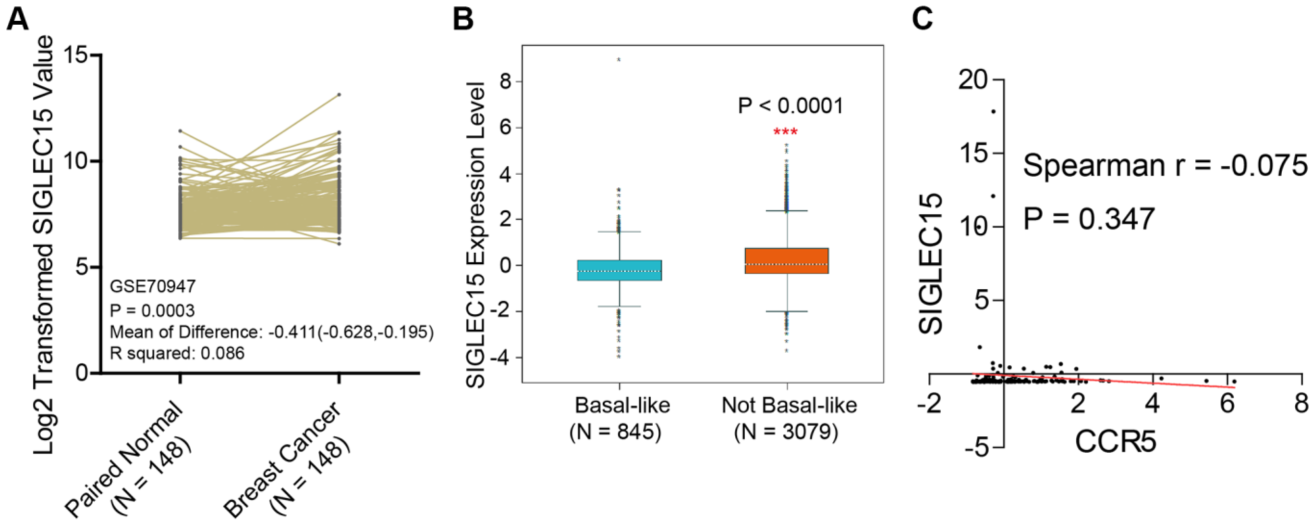

Supplementary Figure 8. (**A**) SIGLEC15 is overexpressed in breast cancer compared to matched normal control. (**B**) SIGLEC15 expression is significantly lower in basal-like subtype compared to not basal-like subtypes. (**C**) There is no correlation between the expression of CCR5 and SIGLEC15.

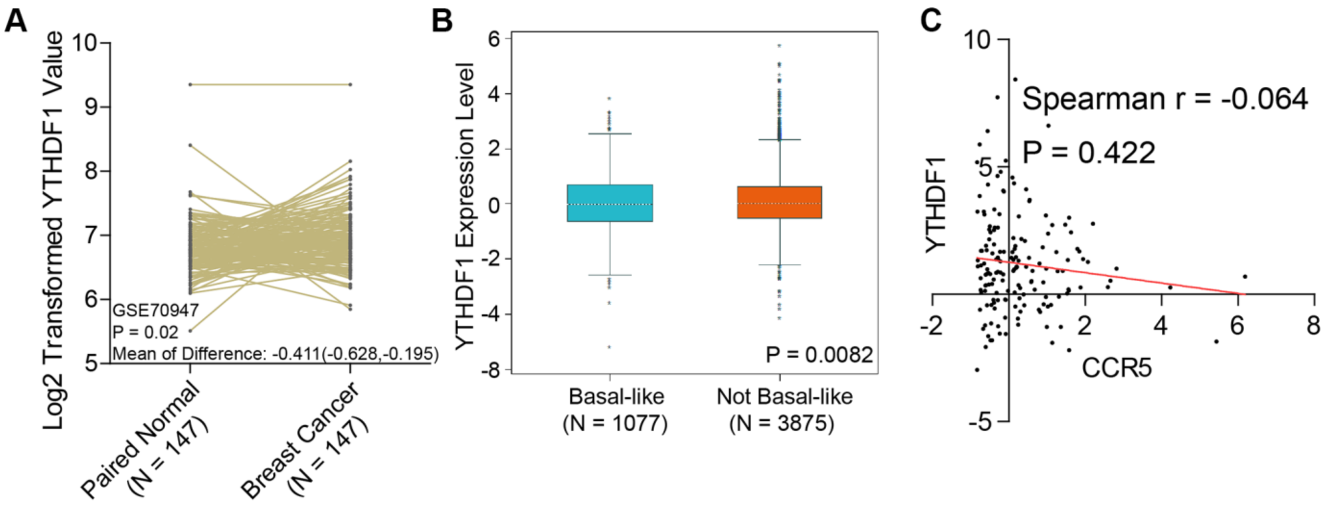

Supplementary Figure 9. (**A**) YTHDF1 is overexpressed in breast cancer compared to matched normal control. (**B**) YTHDF1 expression is significantly lower in basal-like subtype compared to not basal-like subtypes. (**C**) There is no correlation between the expression of CCR5 and SIGLEC15.

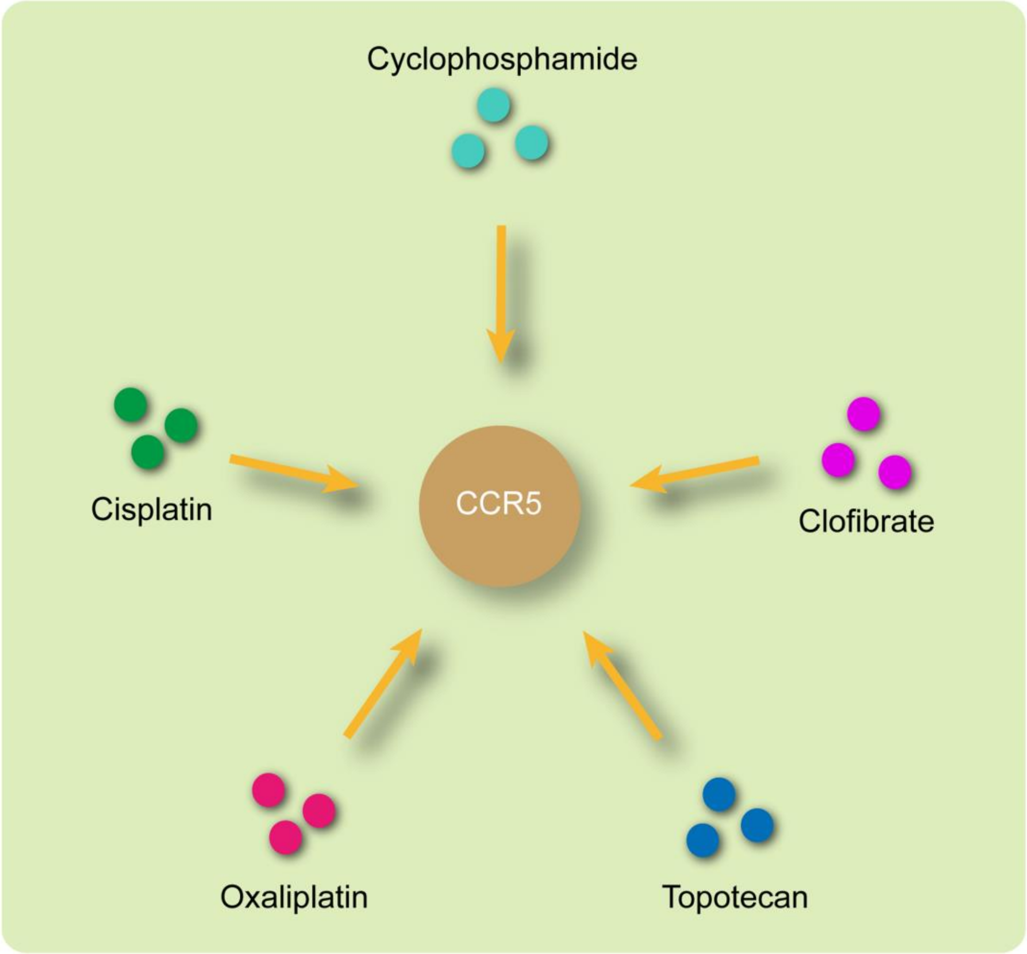

Supplementary Figure 10. Potential drugs that could up-regulate CCR5 expression.
